# Memoirs of the Medical Society of Observation, of Paris

**Published:** 1838-07

**Authors:** 


### Art. II.

*Mémoires de la Société Médicale d'Observation de Paris.* Tome Prem. —*Paris,* 1836.

*Memoirs of the Medical Society of Observation, of Paris.* Vol. I. —*Paris,* 1836. 8vo. pp. 495.

In our last volume (No. IX. p. 154,) we gave a general notice of this volume, and of the principles of the Society from which it emanates. Opposed as we there showed ourselves to the inconsiderate extension of the numerical method to the practice of medicine, we bore testimony to its great value as applied to those departments of the science which rigidly admit of calculation. As further evidence of the sincerity of our opinion, we have carefully analysed the three principal memoirs in the volume, in order that we may make our readers acquainted with their very important contents. We think that, if any proofs were wanting of the benefit conferred on medical science by the more rigid enforcement by M. Louis of the inductive method in medico-statistical enquiries, the memoirs now before us would supply them.

### I. *Recherches sur l'Emphyseme des Poumons.* Par M. Louis. (*Researches on Emphysema of the Lungs.* By M. Louis.)

Although these Researches bear the name of M. Louis, they are not, as he himself informs us, founded on observations exclusively his own. A share of the cases was furnished by the late Dr. Jackson, of Boston, to whose brief and laborious career we have already alluded in this Journal as affording a strong illustration of the power possessed by Louis of infusing a portion of his zeal into those with whom he is brought into contact. That there are "minds fitted," as an eloquent writer expresses it, "to make their spirit pervade and elevate the smaller minds around them," none can doubt who have had an opportunity of visiting the wards of Louis. It is his example and influence that explain the patient toil with which numerous young men there pursue what must be admitted to be, unless with a view to its ultimate utility, the most irksome of occupations—bedside observation. But it may be said there is nothing uncommon in this, and that all men, separating in their doctrines from the multitude, have had ardent disciples among the young. True: but there is a wide difference between the sacrifice of personal ease required in practically espousing the method of Louis, and that of any other great medical innovator. Let us take, for example, the case of Broussais. We do not deny the extent to which his dogma found favour among medical men, especially in France, or the species of *acharnement* with which it was defended; but this attachment is easily explained by the encouragement doctrines like his give to indolence, and by the simplicity of the practice taught in them, which saves its disciples from the annoying process of reflecting on the nature of the cases submitted to them for treatment. There is no need to turn for its explanation to the influence of a master intellect, or to devoted love of science and abstract truth: but how else can the ardour with which Louis' method is followed up be accounted for?—a method in which that mental exercise of all others most pleasing to the young, indulgence in hypothesis and speculation, is as it were proscribed; in which the labour of hours is sometimes required to substantiate a single fact, while that fact is in itself looked on as valueless unless compared with a long series of similar ones!

Whether considered with regard to its gravity and frequent occurrence or to the interest attached to the rise and progress of our knowledge respecting it, Pulmonary Emphysema claims a prominent place in the catalogue of human diseases. Originally mentioned, in so far as its anatomical characters go, by Bonetus, Morgagni, Ruysch, and our own admirable Baillie, it was never methodically studied until Laennec described its symptoms, established its diagnosis, and paved the way for its acquiring the nosological status it now unquestionably holds. Created as its history was by that great observer, in everything relative to this affection we stand on ground almost peculiarly French. Yet even in France, it is far from being generally studied with the care its importance calls for. For while, in the wards of Louis, its symptoms and constituent lesion are examined and demonstrated with the minute accuracy belonging to his school, there are some hospitals in Paris where its name is never mentioned, and others where its very existence is steadily denied. It shall be our business to extract such particulars from the mass of evidence in the conclusive memoir, whose title heads this article, as shall convince the few who remain incredulous that pulmonary emphysema is indeed an entity.

The number of cases analysed in M. Louis' essay amounts to ninety. Of these forty-two relate to subjects who died in the hospital; the remainder to individuals who left it more or less relieved. Twenty-three of the fatal cases occurred from cholera.

The general description of the disease, defined by our author "a dilatation of the pulmonary vesicles," is a summary of the various facts dwelt upon at large in the pages that follow. It is therefore unnecessary for us to extract any part of it, but we take the opportunity of pointing out in it a model of the style in which a general description should be drawn up. Concise without obscurity, it contains everything important, and is loaded with none of that irrelevant matter in which systematic writers are so prone to indulge.

The extent to which the lungs were affected varied considerably in M. Louis' cases, as the following table shows:

| | | |
|---|---|---|
| General emphysema of both lungs in | . | 12 cases. |
| — — of the left lung | . | 2 |
| — — right ditto | . | 1 |
| Emphysema of left upper lobe | . . | 2 |
| — right inferior ditto | . . | 2 |
| — of middle two-fifths of the right lung | . . . | 1 |

These calculations refer to the patients who died of other affections than cholera. In the victims of that epidemic, in whom the extent of the emphysema is also tabularly given, it was general in one-fourth of the cases; whereas, as appears from the above table, two-thirds of the other patients had both lungs emphysematous: this difference is to be explained by the facts that the mean age of the former series of subjects was only fifty, of the latter sixty; and that the extent of emphysema, as of chronic affections generally, is proved to be in the direct ratio of its duration.

M. Louis concludes that "very probably" both lungs are equally subject to dilated vesicles, and that in both the lesion is disposed to attain the same degree of development. He is borne out in this conclusion as regards the *entire* lung by the table from which he draws it; but it ceases to be true if applied to the corresponding parts of the two lungs.

Thus we find emphysema of the left inferior lobe noted in fifteen cases, of the right inferior in two only. And, by adding together the *partial* and *general* cases for each lung, we find forty-seven the total for the left, and thirty-four for the right. Where the elements for calculation are so limited, this surely is no very trifling difference. The difference in the comparative frequency with which the inferior lobes on both sides are attacked is extraordinary enough, yet M. Louis indulges in no comment upon it.

Whatever be the degree of dilatation of the vesicles it is never uniformly the same in the different parts of the lung. The internal surface and base of the organ usually present the largest cells; and in fifteen cases in which their relative dimensions were carefully noted, those of greatest size were found at the acute border. From this fact M. Louis, as we shall learn by and bye, draws a pathological inference of primary importance.

Our author frequently alludes to certain *appendices*, as he names them, probably answering to the sub-plural vesicles of Dr. Stokes, which constitute the most advanced stage of the emphysematous lesion. The post-mortem details of the first case reported in his essay contain a very close description of these.

"The form of the *appendice* was renal, its length four inches, its breadth one and a half. When dried and cut across at an inch distance from each of its extremities its structure was found to be as follows: one of the sections presented, at its centre, vesicles smaller than a millet-seed, circumscribed by transparent and extremely thin septa; these cells increased in size the nearer the surface they were examined. Here they were as large as a hempseed, or larger, their shape irregular, oblong, and, as it were, denticulated. The other segment of the *appendice* was somewhat larger, contained three principal cavities of the size of a nut, traversed by cellular filaments; besides smaller cells separated by lamellæ of exceeding thinness, shining like the coats of an onion. They were from three to four lines long, by very nearly the same breadth and crossed by several vessels, scarcely to be recognized by their colour." (p. 175.)

In this case, although the disease existed to a very marked extent, the difficulty of breathing was far from excessive.

In thirteen cases the bronchi were carefully followed to their terminations, and in four instances only were they found dilated. The smallness of this number of dilatations excludes, according to M. Louis, the idea of community of affection between those tubes and the vesicles, inasmuch as "it is perhaps not greater than that existing under different circumstances in subjects of the same age as those in question." We should have been inclined to consider it materially greater, but even if we were correct in this supposition, and we admit we are without the only true elements of a positive decision, namely, the examinations of the bronchi in a large number of patients dying of various diseases; still the following facts, brought forward by our author, prove that the bronchial and vesicular dilatations are in his cases independent of each other. First, the dilatation of the bronchi was confined to a small extent of lung, where that of the vesicles was general. Secondly, in two cases in which the disease had advanced to the formation of *appendices* the bronchi were not dilated; in one of them they appeared even of smaller caliber than natural. Thirdly, when both lungs were emphysematous, the dilatation of the bronchi was almost always confined to one side. Fourthly, though the vesicles were most enlarged near the acute border the bronchial dilatation did not affect any preference for that part of the lung.

The pleura was affected with adhesions in thirty out of thirty-six cases; but our author is induced to deny any influence to the emphysema on the development of these lesions on several grounds, which we shall give in his own words.

"In the first place universal adhesion of both lungs did not exist in a single case, and general adhesions of one side were only found in fifteen individuals. In the others they were confined to a single lobe and frequently to an extremely limited portion of the pulmonary surface. Hence, as regards pleural adhesion, emphysematous patients are found to be almost similarly circumstanced to non-tuberculous subjects unaffected with emphysema. To this must be added that the individuals whose cases are under consideration, were generally of a more advanced age than the aggregate of those who die of any given disease. This fact accounts for the trifling difference observed, for the general causes of pleurisy had been longer in action in the former than in the latter subjects. Moreover the lungs were totally free from adhesion in two of the three patients in whom the emphysematous disease had reached its highest point; and the pleural false membranes, when partially spread over the lung, were found, as is the ordinary case, *posteriorly*, even though the vesicles were most largely dilated at the anterior part of the lungs." (p. 179.)

Tubercles or grey granulations were found in the lungs in a smaller proportion of subjects affected with emphysema than the mean number found in individuals dying from various other diseases: this result is corroborated by many minor facts brought forward in the memoir.

We fully agree with M. Louis in the justness of the inferences he draws from these positions. They prove that pleuritis, tubercles, and emphysema do not stand to each other in the relation of cause and effect. They are also a pointed illustration of the capital importance of studying the state of every organ in large masses of subjects dying of all varieties of disease, before we can presume to decide on the reality of a supposed connexion between any coexisting lesions.

The heart was hypertrophous in sixteen cases. It had acquired double its natural size in one, and exceeded that degree of enlargement in two subjects. Six of these cases occurred in the twenty-three cholera patients, ten in the nineteen remaining subjects. It would seem natural to ascribe this difference simply to that in the mean ages of the two series of individuals. Nevertheless, M. Louis conceives it in some measure attributable to the emphysema "because the excess in the heart's size was much less in the cholera patients, in whom the dilatation of the vesicles itself was generally less advanced, than in the other subjects." Our author does not inform us which side of the organ was principally affected or whether both were equally so.

The second section relates to the symptoms. Dyspnœa, the most constant of these, as it existed in forty-two out of forty-four cases, first occupies M. Louis' attention. We may observe that in the two exceptional cases, in which neither the reality of the emphysematous disease nor the absence of dyspnœa can be questioned, there was no habitual pulmonary catarrh. Our author is disposed to believe that it is only in cases where the affection is of short standing that dyspnœa is wanting; an idea which to a certain extent is in opposition with the fact that atrophy of the vesicles, the most advanced stage of the lesion, coexisted in a case above alluded to with easy respiration. The difficulty may, it is true, be got over by supposing a very rapid development of the disease; but this, as we shall see, is, at the least, a rare occurrence.

M. Louis' details on this single symptom fill six pages. The following is an outline of the principal facts contained in them. 1st. The oppression was persistent in every instance, except in one individual aged eighteen, who was not subject to cold. The physical signs of the disease were all of them clearly made out both during and after the paroxysm of dyspnœa for the relief of which this patient came to the hospital. 2dly. The period of origin of this symptom varied exceedingly. In sixteen subjects it existed from the earliest infancy. In the others it commenced as follows: before the twentieth year in two patients, from the twentieth to the thirtieth year in seven, from the thirtieth to the fortieth in seven, from the fortieth to the fiftieth in eight, and from the fiftieth to the sixtieth in three. No cases were met with after that age, which seems to show that the *formation* of emphysema is scarcely to be feared at so advanced a period of life, though its *increase*, when previously developed, is then frequently met with. 3dly. The difficulty of breathing was usually slight in the outset and increased in some cases uniformly, in others irregularly, at a period more or less distant from its commencement. 4thly. In the majority of cases, thirteen out of seventeen, this increase in the dyspnœa ushered in another change, viz. habitual dyspnœic [asthmatic] paroxysms. These exacerbations were generally mild in patients whose dyspnœa commenced in early youth. 5thly. In many cases the exacerbations came on without apparent cause; they were, however, usually induced by an attack of acute pulmonary catarrh. 6thly. It is not known if atmospheric variations have any influence on their production; but if they have such influence, it is certainly not constant in its action. 7thly. The dyspnœa of emphysema is in itself alone exceedingly characteristic of the disease. It could not, in M. Louis' cases, be due to habitual bronchitis,—because that affection did not exist in every case, and because it only commenced in infancy in one solitary instance; nor to dilatation of the bronchi, as the dyspnœa, which is sometimes observed in that affection, is slight and not attended with exacerbations; nor to disease of the heart,—because in the cases in which a morbid state of that viscus was observed, far from having originated in youth, it had only existed a few years; nor to phthisis,—on account of the absence of hæmoptysis and other tubercular symptoms.

It is plain that the differential diagnosis is not in the last case, at least, founded singly on the character of the dyspnœa; and the following facts, discovered by the late Dr. Jackson, while they prove the vast diagnostic value of the symptom, show that it cannot be considered perfectly pathognomonic of emphysema. Of one hundred and twenty subjects, the state of whose respiration was carefully ascertained from infancy upwards, twenty-eight had been subject to dyspnœa from that period: of these twenty-eight individuals, one was affected with cardiac disease, two with phthisis, *twenty-five* with pulmonary emphysema.

There is a point of some importance noticed relatively to a case introduced in this part of the memoir. The subject of it had intercurrent pneumonia. After the resolution of the inflammation, there was no sensible increase in the dyspnœa symptomatic of the preexisting emphysema. "Hence it appears," says the author, "that we cannot with certainty of being correct attribute emphysema supervening shortly after pneumonia to that disease," (p. 192.)

The alterations produced in the form of the chest by dilatation of the vesicles, are among the most remarkable effects of the disease. In one case the entire thorax was altered in shape, having acquired a globular form. This general prominence depended at once on the altered position of the ribs "and of the intercostal spaces *which were not sunk,* as is, especially in thin subjects, their natural condition," (p. 195.) The change of form in the remaining cases, thirty-six out of thirty-seven, the only ones in which it was examined, was of precisely similar nature, but only partial. It usually extended from the clavicle to or even beyond the mamma, and measured from three to four inches in width. The great diagnostic importance, in M. Louis' opinion, of this prominence will appear from the following considerations: It cannot be mistaken for a congenital malformation, *because the ribs and intercostal spaces concur equally in its formation;* nor confounded with the result of pleuritic effusion, because in this the dilatation is general, and more marked inferiorly than superiorly; nor with an aneurism of the aorta, because the prominence produced by that affection is more circumscribed and more elevated. It might be confounded, on simple inspection, with the arch caused by effusion into the pericardium or hypertrophous heart, but percussion would of course at once remove all chance of error.

According to Dr. Stokes, "the intercostal spaces in this disease *are deeply marked, and present no indication of protrusion.*" Here is a direct contradiction of the result of Louis' experience. We acknowledge our inability to reconcile statements so utterly opposed to each other: but, in justice, we must add that our own acquaintance with the signs of the affection induces us to believe M. Louis correct; and we are confirmed in this idea by the results at which M. Woillez arrived in examining a collateral question. In the essay of that gentleman on the physiological and pathological prominences of the chest, founded on the careful measurement of one hundred and sixteen thoraxes, we find the following proposition. "If there exist a *physiological* prominence at a given point of the chest, and the part of the lung answering to it grow emphysematous, that prominence may be considered to have become *pathological* as soon as the *intercostal spaces are effaced;* but previously, while those spaces are still distinct, the elevation cannot be confounded with those produced by emphysema," (p. 53.)*

The prominence described was not observed with equal frequency on both sides of the chest. It occurred in eleven cases on the right side, in twenty-three on the left. This result, as the reader will perceive, is in opposition with a statement already made to the effect that the frequency and degree of the disease are equal on both sides. M. Louis endeavours to escape this difficulty, by supposing the inequality as to change of form only temporary, and likely to disappear in proportion as the development of the disease advances. The fatal cases do not, as far as we can perceive, favour his hypothesis.

A far more plausible solution of the difficulty has been proposed by M. Woillez. It appears, from the numerous measurements of that gentleman, that *physiological* prominences of the anterior surface of the chest are almost always found on the *left* side, and are tolerably frequent there. "Are we to believe then," he asks, "that the *morbid* prominence of emphysema is, in the outset, frequently *physiological;* or that the unknown cause, which favours the formation of physiological elevations on the left side, acts also on that of the pathological dilatation of the thorax produced by emphysema?" (*Op. cit.*, p. 37.) We should be inclined to go even further, and hazard a conjecture that some of the *left* prominences, observed by Louis, were solely physiological, and uninfluenced by the subjacent pulmonary lesion. An evident consequence of M. Woillez's discovery is, that, though occurring much more frequently at the *left* side, the prominence in question is of much more value, as a diagnostic mark of emphysema, when found on the *right.*

It is clear that explanations such as those suggested above, need only be had recourse to if the presumed equal liability of both lungs to the disease really exist. Now we have shown that, even from the evidence of M. Louis' cases, it is far from being satisfactorily proved to do so. And it is curious that the quantities representing, on the one hand, the number of times each lung was affected, and, on the other, the number of times each side was partially dilated, namely 47, 34, 23, and 11, are extremely close to the true proportionals as 47 : 34 : : 23 : $16\frac{30}{47}$.

The back of the thorax was only examined in six cases,—evidently an afterthought,—and in three of these, a partial dilatation was discovered. We can scarcely understand how, in accordance with his principles, M. Louis justifies himself in concluding, as he does, from data so limited as

* Thèse sur la valeur diagnostique des Déformations de la Poitrine, &c.: one of the most interesting monographs, though only an inaugural essay, that we have for a long time met with. M. W., we see, has since published a book on the subject, which we shall notice hereafter.—Ed.

these, that "the emphysematous prominence appears to affect a preference for the anterior surface," (p. 196.) He tells us, it is true, that such a fact would be in harmony with what has been proved respecting the anterior border of the lung being the usual seat of the largest vesicles. No doubt it would. But is this *direct observation?* Would not M. Louis reject with disdain any such argumentation on the part of another in favour, for example, of the inflammatory origin of tubercle? We think he would,—we are certain that he ought. The researches of M. Woillez have, as it happens, shown that M. Louis' *à priori* notion was correct, but he cannot defend his departure from his principles by this *lucky chance.*

Another prominence seated in the post-clavicular space, and originally discovered by our author, usually coexisted with the mammary dilatation. Its frequency was so great that it was found in every case but one, after its first discovery. It is especially remarkable in old meager subjects, in whom the region referred to appears to have regained its youthful fulness of form. As M. Woillez judiciously remarks, the state of the post-clavicular region furnishes a very useful guide in cases where the physiological or pathological character of the mammary prominence is a matter of doubt.

Again, Dr. Stokes' experience differs completely from that of Louis. After stating in his recent work that he had seen several cases in which the acromial, interscapular, supraspinous, and subspinous surfaces had become nearly horizontal, he continues: "Under these circumstances the apices of the scapula are remarkably projected; anteriorly we observe the clavicles arched and prominent; and the *triangular spaces which answer to the insertion of the sterno-mastoid and scaleni muscles are singularly deep,*" (p. 177.) Further observation must decide between these two eminent pathologists; meanwhile we may be permitted to state, that we have not, in a considerable number of cases attentively examined, met with a single instance of the appearance described by Dr. Stokes.

One important fact was elicited from the post-mortem examination of the fatal cases, the correspondence of the thoracic dilatation to the portion of the lung most advanced in disease. M. Louis enters into no enquiries as to the mode of production of the emphysematous prominence, a point of much interest, and one on which M. Woillez has given us some reasonings closely logical and of rare ingenuity. We regret our limited space prevents us from laying them before our readers.

The next symptom examined is the quality of the sound emitted on percussion over the affected parts of the lung. On this subject we are given no information of sufficient novelty to merit notice. The author judiciously insists on the diagnostic importance of a combination of anormal clearness of sound, in any point of the thorax, with dilatation of its walls. As might be anticipated, the disease, when confined to the internal surface or central part of the base of the lung, cannot be detected by percussion. Our author met with one such case.

M. Louis' remarks on the condition of the respiratory murmur are interesting. The feebleness of respiration characteristic of the affection was ordinarily confined to a limited extent of the chest, rarely spread over its entire surface. This character of the breathing persisted unchanged during the entire stay of the patients at the hospital, except in the instance of one individual in whom it decreased considerably. "In four cases the respiratory murmur was *slightly* hard, as though produced by the entry of the air into a much smaller number of cells than on the opposite side, where the respiration appeared finer and softer," (p. 212.) As these characters of the respiratory murmur are precisely those observed in dilatation of the bronchi, a doubt seems naturally to arise as to the propriety of attributing them to emphysema. M. Louis removes it satisfactorily, by informing us that there was no bronchophony in the cases alluded to. This feebleness of murmur was generally proportional to the duration of the disease, but the rule in this respect suffered some notable exceptions. In three subjects more particularly, in whom the affection had only existed from two to two and a half years, it was more marked than in some others that had laboured under it for ten.

M. Louis observed two kinds of rhonchus in his patients, the sibilant or sonorous and the subcrepitant. The former existed in thirteen subjects out of thirty-one. Usually general, it was in four cases limited to the prominent part of the thorax. Our author considers the latter fact to indicate the possibility of its having some special connexion with emphysema.

The study of the subcrepitant rhonchus possesses more interest, though, as we shall perceive, it is completely independent, according to M. Louis, of the emphysematous lesion *per se*. It existed in twenty-two of the forty-eight subjects who left the hospital relieved. It was characterized by being seated at the postero-inferior part of the chest, in the majority of cases at both sides, and was never detected in the mammary prominence. Nevertheless there is no necessary incompatibility between it and the enlargement of the vesicles. In many cases in which the rhonchus was audible posteriorly, dilatation of the cells certainly existed, according to our observer, in the same position. But the truth is that the sub-crepitant râle occurring with the characters described, has been shown by M. Louis to be a pathognomonic sign of acute capillary bronchitis, and, in the cases under consideration, simply proved the presence of an intercurrent attack of that affection. The existence of this râle at the time of entry, and its gradual decrease in proportion to the relief experienced by patients during their stay at the hospital, shows that acute pulmonary catarrh is the *immediate* cause that forces emphysematous subjects to apply for medical aid.

M. Louis conceives, and we think to a certain degree on just grounds, though from the vagueness of Laennec's statements it would be difficult to prove the fact, that this râle is the identical one pointed out by that observer as pathognomonic of emphysema. The facts above adduced, showing that the subcrepitant râle of emphysematous patients follows the same laws as that of individuals exempt from that disease and affected with *simple* catarrh only, render it evident that the error lies mainly with the latter pathologist. But we do not wholly agree with M. Louis either. We have ourselves frequently heard, and pointed out to others, in the situation of the mammary prominence, minute bullæ of dry subcrepitant rhonchus, a medium sound between the *croquement* of the French and the *bruit de frottement*, and this at a time when there was no râle audible at the base posteriorly, or any other symptom present of pulmonary catarrh. It is just possible that these sounds are what M. Louis describes as "hard respiration," but, if so, his account of the phenomenon falls, in our estimation, far short of the reality.

We regret that our author's enquiries on the physical signs of the disease are confined to those of which we have just taken a rapid survey. He makes no remarks on the distinctive characters of the inspiration and expiration; he does not specify with which of them the rhonchi coexisted or if they attended both, nor does he allude to the altered duration of the time naturally elapsing between the two movements; yet these are points capable of accurate discrimination and, as we can from experience testify, likely to furnish a pains-taking observer with many curious diagnostic data.

The state of the motions of the chest and diaphragm are equally passed over in silence.

Some observations are made on pain of the chest as attendant on emphysema that strike us as novel. This symptom was noted in fifteen out of thirty-two cases, and in thirteen of these it was situated on the same side as the thoracic dilatation. It was increased by inspiring or coughing, but M. Louis was unable to determine its other characters; a strange fact, when we reflect that it had existed from one to three or four years and upwards, in the instance that fell under his notice. Rejecting the hypothesis of its being caused by pleural inflammation, or by the forced extension of the walls of the chest, he is disposed to ascribe it, admitting the want of demonstration, to the dilatation of the vesicles themselves. Several circumstances are favorable to this view of its cause: among them we may instance the correspondence of the principal seat and chronic course of the affection with the location and duration of the pain, together with the improbability that so extensive a dilatation as the cells sometimes undergo, should take place without exciting uneasy sensations.

The frequency of cough in these cases renders it a valuable symptom. Variable in its intensity and time of origin, persistent or intermittent, it was observed in every case but one. In twenty-two cases it commenced at the same time as the dyspnœa, fourteen times after and ten times before that symptom. Whether continuous or intermittent, it never commenced at the same time as the dyspnœa when the latter supervened in early youth, and did not appear until after that symptom, in cases in which it manifested itself *for the first time* after the age of twenty. The bearing of these facts militates to all appearance rather strongly against M. Louis' correctness in fixing infancy as the time of origin of the disease in subjects whose dyspnœa commenced at that period. He succeeds, however, in showing himself authorized in doing so, but his arguments are too long to admit of insertion here.

The sputa were carefully examined in thirty-five subjects with the following results: " In twenty-three cases they were frothy, aërated, or liquid, resembling a solution of gum; in the remaining twelve subjects greenish, thick, opaque, but little aërated, and not in rolled masses; or greyish, with some striæ of blood," (p. 228.) The former sort of expectoration was observed in individuals affected with chronic pulmonary catarrh, producing sibilant or sonorous rhonchus; the latter in patients labouring under the same complaint in the acute stage, and presenting subcrepitant râle at the base posteriorly. Our author notes as a new illustration of the necessity of a *special* cause for the production of hæmoptysis, that, of these thirty-five individuals, only one, a female, aged fifty-seven, who had ceased to menstruate at the age of twenty-seven in consequence of a sudden fright, had suffered from that symptom. Our readers are no doubt aware that, according to one of M. Louis' laws, severe hæmoptysis is peculiar to individuals affected with pulmonary tubercles.

The state of the heart was examined into in fifty-two subjects. Thirty of these were found to be more or less troubled with palpitation. In some that symptom only came on after fatigue or during the asthmatic paroxysm, in others its occurrence had no connexion with either. With the exception of individuals in whom they were of temporary duration, the palpitations did not commence at the same period as the dyspnœa, and generally made their first appearance after the disease had half run its course. The volume of the organ was found to be greater than natural in subjects in whom its irregularity of action had been continuous. M. Louis hereupon alludes to the difficulty of ascertaining the exact state of the heart by percussion or inspection of the præcordial region, in consequence of its increased sonorousness and occasional arched form.

Enlargement of the heart was discovered on the dissection of every patient who had during life laboured under œdema of the inferior extremities; while, in not one of those unaffected with œdema, was the heart hypertrophous. Hence M. Louis' conclusion, that "œdema supervening in the progress of pulmonary emphysema must be ascribed to its complication with organic affection of the heart," (p. 232.) The diagnostic importance of this proposition is evident.

Several pages are occupied with considerations on the diagnosis, but as they are in the main a mere recapitulation of the more important passages that precede, we are dispensed from fully analysing them. To one or two points only it may be useful to advert. One of these relates to the distinction of emphysema from aortic aneurism. A case of the latter disease was met with by M. Louis in which there was no thoracic prominence, while the respiratory murmur was inaudible or almost so in a considerable part of one side of the chest (a phenomenon noticed some time past in the same disease, if we mistake not, by Dr. Stokes,) and percussion produced a perfectly clear sound.

The case is an important one from its showing that a combination of perfectly clear sound and absence of respiratory murmur are not pathognomonic of emphysema. The absence of respiration is easily explicable by the pressure of the aneurismal tumour on the larger bronchi; but we cannot acquiesce in M. Louis' conviction, that the absence of thoracic prominence would suffice for the distinction of the two cases. What is there to prevent our supposing the anormal state in question to be the result of *incipient* emphysema, in which the thoracic walls had not yet had time to give way? Nay more, of *advanced* emphysema,—for M. Louis has nowhere proved that the development of the disease involves the necessity of a change in the proportions of the chest; and M. Woillez has, in the thesis already quoted, clearly shown that such necessity does not exist. In the very same page, indeed, our author himself testifies to the force of our objection. After stating that all the symptoms described, with the exception of those announcing cardiac complication, existed in almost every case observed by Dr. Jackson and himself,—an important fact,—he proceeds to show the diagnostic value of different groups of them when others were absent. Now he commences with the case in which no partial prominence existed.

There is one view in which the power of diagnosticating emphysema is an acquirement of high value to the practical physician. It enables him to dispel the fears of many patients in whom the disease, from the habitual oppression and bronchitis accompanying it, assumes to a certain extent the character of pulmonary consumption. It warrants him, too, in exposing the quackery of those unprincipled men who, aided by the natural credulity of suffering patients, pretend, in the teeth of all scientific experience, to have the power of curing pulmonary tubercle.

An interesting fact is here brought forward connected with the coexistence of tubercles and emphysema. In his Researches on Phthisis, after stating that dyspnœa from infancy was ascertained to have existed in one-ninth of his tuberculous patients, and deciding that it could not be ascribed to the tubercles themselves, M. Louis deferred the explanation of the fact to a future day when some discoveries in pulmonary pathology might throw light on the difficulty. The presence of emphysema, a disease which our author had scarcely studied when he published his book on phthisis, was beyond a doubt the cause of the dyspnœa in the consumptive subjects alluded to. A precisely similar case is given in the present essay.

A sketch is drawn of the history of a patient, affected with both diseases, in whom the physical signs of emphysema gradually disappeared, while those of tubercles increased. This change, the natural result of the growth of the latter, explains how an observer might have been perfectly right in announcing the presence of emphysema in some cases, where after death scarcely a trace of the disease was discernible The lapse of time required to bring about so material a change in the pathological state of things may, in cases of acute phthisis, be far from considerable. A case is also reported that seems to prove satisfactorily that extensive enlargement of the vesicles may take place in so short a time as twenty-nine days.

The remarks on the causes of the affection are limited in extent, and chiefly of a negative kind. Let it not be supposed, however, that we undervalue information of this kind. The following facts prove in M. Louis' opinion, that if bronchitis have any influence on the production of the disease, it must be very trifling and rarely brought into play. In our minds they show that Dr. Stokes has gone too far in styling emphysema "the result of bronchitis." First, in numerous cases the symptoms of dilated vesicles are not preceded by those of catarrh. Secondly, the habitual dyspnœa did not appear, in several instances, to suffer any increase after an attack of acute bronchitis. Thirdly, the maximum degree of the disease is found at the anterior border of the lung, while acute catarrh is most fully developed at the base posteriorly. A fourth argument on the same side may be found, as it seems to us, in a fact discovered by Dr. Stokes, and of which he appears to have overlooked the bearing on this question. He says, "the sign of feebleness of respiration is but little affected by the increase or diminution of bronchitis." Now, the increased volume of the lung, which, according to Dr. Stokes'

views, causes the feeble respiratory murmur, is produced by dilatation of its vesicles, consequently so must the diminished force of respiration be the effect of that enlargement. But as the respiration is little affected by bronchitis, it follows that the dilatation of the cells, which is proportional to the weakness of that respiration, must be but little affected by it also. In general terms this is tantamount to saying that a cause which is powerful enough to produce a disease, (as inflammation of the bronchial tubes, according to Dr. Stokes, produces emphysema,) loses the capability even of influencing its symptoms when it has once brought it into being. Such a proposition as this cannot surely be assented to.

Laennec's well known hypothesis as to the mechanism by which the dilatation is immediately brought about, though sufficiently plausible, seems certainly little shaken by M. Louis' observation that, " whatever be the size of the enlarged vesicles, be it even that of a cherry stone, they are found empty, without either mucus or false membrane in their interior," (p. 254.) We have already, in our Number for last October, p. 311, alluded to M. Louis' objection, and stated our reasons for not completely giving in to it.

As regards the question of hereditary influence, Dr. Jackson's patient enquiries furnished him with the following conclusions: " Of twenty-eight emphysematous subjects eighteen were of parents who had suffered under the same disease, many of them having died during its progress. In some cases the brothers and sisters were similarly affected. Of fifty individuals unaffected with emphysema, three only were of parents that had laboured under the disease," (p. 255.) Pulmonary emphysema is by these facts clearly proved to be frequently an hereditary affection. They are corroborated by another series of facts discovered by the same zealous observer, showing hereditary influence to be much more marked in cases where the disease commences in early youth than where it first appears towards the twentieth year.

Of the various conclusions deducible from our author's pages, that for which the reader was possibly least prepared is the evidently great frequency of the disease described in them. The greater part of the cases were observed in the space of twenty months, in wards containing ninety beds. The large proportion of emphysematous subjects among the victims of cholera, twenty-three out of fifty, affords still further evidence of its remarkable prevalence.

M. Louis' section on treatment is exceedingly brief and unsatisfactory. As prophylactic means, he advises the avoidance of all known causes of diseases, of those of the lungs in particular. With respect to the therapeutic effects of polygala, the oxymels, medicinal soap, &c., which are earnestly lauded by Laennec, our author is disposed to deny their reality altogether. According to him, the amelioration observed by that physician during their exhibition resulted from repose, hospital diet, and use of diluent drinks. We confess that having ourselves seen the good effects of this negative treatment in the hands of M. Louis, we find it difficult to gainsay his statement. Nor is the opinion he has formed of the efficacy of bleeding, either in serious or slight cases, of a more favorable character. His confidence in the various preparations of opium is on the other hand very great. In twenty-six cases out of thirty, notable relief immediately followed its exhibition, an improvement which could be attributed only to its use, as the asthmatic paroxysm recovered its primitive intensity the moment it was, by way of experiment, discontinued. It is in the most severe cases, where the exacerbation is violent, and the presence of capillary bronchitis proved, that the excellent effects of opium are seen to the fullest advantage. Among the cases alluded to in support of its efficacy, there is one that too clearly shows its superiority to venesection as a remedy in this affection, not to deserve extraction. The patient to whom it refers "was bled three times in the course of two days without experiencing the slightest benefit, the dyspnœa remaining unaltered, and a fatal termination appearing more imminent after the third than before the first bleeding. Recourse was then had to the gummy extract of opium, which was given to the extent of two grains in a few hours. On the following day the dyspnœa was very moderate, the respiration much less frequent, and the patient so materially relieved that he considered himself cured," (p. 260.) In some similar cases, occurring subsequently to this one, no bloodletting was employed and the opium exhibited at once. The relief in this way was no less prompt and complete.

The cases interspersed through the memoir, which are only six in number but all of much interest, confirm, as far as their limited evidence goes, the general propositions of the essay. Their bearing on each question is carefully pointed out by the author, nor have we detected any instance in which more stress is laid on them than they seem to warrant.

On the whole, these researches will form a valuable addition to the libraries of those who are studious of correctness in diagnosis, and had they come from an ordinary observer would justly have been esteemed a very remarkable performance. As it is, they probably may not detract from but certainly do not add to their author's claims to our respect. They want that searching sagacity in induction that abounds in his other works, and their most valuable propositions are mainly deduced from knowledge—original and his own, it is true—but still previously acquired. M. Louis is indeed his own severest critic, for by what standard but his own labours should the author of the "Recherches sur la Gastro-Entérite" be measured? Assuredly none. In that almost unrivalled work, M. Louis has given evidence of the strongest faculties for observing, of which the mind may be believed capable, and seems, while he has produced results that will be more and more valued in proportion as men learn to appreciate the excellence and grandeur of inductive truth, to have reached the limits of probable perfection.

II. *Recherches sur le Cœur et le Système artériel chez l'Homme.* Par J. Bizot, de Genève, m.d. &c. (*Researches on the Heart and the Arterial System in Man.* By J. Bizot, m.d.)

The memoir of M. Bizot consists of a series of minute investigations, from which general conclusions are drawn by the numerical method, on certain points of the normal and morbid anatomy of the heart and arteries. It may, on first view, perhaps, appear that a work on this subject was unnecessary, and especially uncalled for, just after the publication of M. Bouillaud's bulky treatise. An analysis of its contents will, however, we trust, convince our readers that the facts established in it are of the most part new, and peculiarly worthy of attentive study, from the accuracy and extent of the data on which they are founded.

The number of individuals examined by our author in the prosecution of his researches amounted to 157 : of these, (of whom an equal number belonged to each sex,) 122 were upwards of, and 35 under, fifteen years of age. Considerable as this number of facts is, we conceive M. Bizot is not over scrupulous in viewing them as insufficient to furnish definitive laws.

The method followed by M. Bizot in obtaining the exact measurements of the heart, and accurate notions of its lesions generally, was the best calculated for the avoidance of error. As a proof of his determination never to sacrifice perfect accuracy to brilliancy or seeming perfection of results, we may notice the following fact: Finding the irregularity of form of the auricles to be such that a particular preparation of the venæ cavæ would have been required to enable him to judge with exactness of their interior, and, being unable to make this, he leaves the questions of their capacity, &c. open to future observers. It would have been easy for M. Bizot to obtain approximative results from ordinary data; but he felt that "the numerical method, applied to medicine, possesses all the imposing authority and all the dangers of statistics. Legitimately employed, it establishes truths; applied to materials of doubtful accuracy, it may propagate, under the appearance of close demonstration, the most baneful errors. Is it not, then, the bounden duty of the enquirer who avails himself of it to work on a perfectly solid basis, or refrain from its use altogether?"

M. Bizot's opening table proves the following facts: First, that we cannot obtain a *single* standard for the heart's volume, inasmuch as it varies according to the age, sex, &c. of the subject; secondly, it discloses a law of primary importance,—namely, that an indefinite increase in the size of the organ occurs in all subjects in the ratio of their age, and ceases only with life. This law, it must be remembered, is deduced from cases unattended with any symptoms of cardiac disease, and is therefore to be considered one of those regulating the economy in its normal state. The growth is regular and constant in both sexes, as respects length and breadth. In every series of ages, the female heart is smaller than the male; even in very young subjects: a result which coincides remarkably with the observation of M. Quetelet, that female infants weigh less than male infants at the moment of birth. This law of the indefinite growth of the heart is greatly at variance with one of Bichât's opinions. In one of his bold generalizations, he asserted that it is "from the twenty-fourth to the twenty-sixth year the muscles of organic life acquire their full development." Our author's researches satisfactorily prove his error in the case of the heart: careful investigation can alone determine whether the doctrine be less faulty when applied to other organs. The same law shows the inadequacy of the test of the heart's size to the normal state proposed by Laennec,—namely, its correspondence with the size of the clenched hand of the subject. It is hardly worth while to notice how it also refutes the absurd notion entertained by Richerand and others, that individuals, having proportionally the largest heart, are also the most courageous. It will hardly be maintained that animal courage is greatest in old age.

The influence of stature on the heart's size appears to be slight. Such as it is, however, it is exactly of the opposite kind to what we should, *à priori*, be inclined to suppose: for, "in individuals of the male sex above sixty inches, and in females above fifty-five inches in height, the mean dimensions of the organ, especially of its breadth, is less than in persons of lower stature." The difference in males averages upwards of three lines. The width of the shoulders furnishes a better proportionate standard of its various measures: the distance between the acromial point of the clavicles and the length and breadth of the heart increase in tolerably regular ratio.

The information supplied as to the influence of disease of the various organs on the heart's size is rather limited. The labours of a single individual could scarcely, perhaps, be expected to throw light, at least in all its bearings, on so extensive and complicated a question. M. Bizot has, however, collected in one group fifty-seven cases of phthisis, and compared the mean dimensions of the heart in them with similar measurements in sixty-five patients who died of various other affections. The result is that, both in males and females, the organ is smaller in all its proportions in tubercular subjects than in others. As phthisical cases are here compared with all other diseases united, there is no contradiction between the proposition now laid before the reader and the fact already established by Louis, that the heart is smaller in cancerous diseases, especially of the stomach and uterus, than in any other.

The above results, it will be observed, contradict the hypotheses of Laennec, Bertin, and Bouillaud, as to the agency of phthisis in producing hypertrophous heart; while they substantiate the induction of Bayle and Louis as to the independence of these two morbid states.

The general propositions established by M. Bizot relating to the extent of the ventricular cavities are the following: 1, their capacity is constantly increasing; 2, they are broader than long in both sexes and at all ages; 3, the mean length and breadth, especially the latter, of the right ventricular cavity considerably exceed the corresponding measurements on the left side, and the ratio of increase on both sides continues equal at all ages. By the latter fact, we are justified in rejecting as erroneous the opinion of Béclard that, at an advanced age, the right cavity undergoes a proportionally greater increase than the left. The cavities of the ventricles are smaller in females than in males, and in phthisical subjects than in those affected with other diseases.

The mean thickness of the left ventricle is shown to increase regularly with the age of the subject. Béclard is here again proved in error, in asserting that the tissue of the heart grows gradually thinner in advanced age. The same facts also indicate the imperfection of Cruveilhier's standard of hypertrophy,—a thickness exceeding seven or eight lines. Without adverting to the fallacy necessarily resulting from the variations dependent on age, &c., the tables before us fully prove that the walls of a left ventricle, seven lines thick in the male, or six in the female, would be in a commencing state of hypertrophy. The laws affecting the thickness of the walls of the right ventricle differ remarkably from those of its fellow. The age and sex of the subject, the heart's volume, the capacity of the ventricle itself, here exercise very little influence. Hence it appears that "the majority of writers, in taking the right ventricle as the point of comparison in estimating the proportional thickness of the left, have adopted the most defective method that could be selected. The reason of this is plain: the thickness of the right walls remains almost stationary, while that of the left is continually on the increase," (p. 289.)

Judging from M. Bizot's facts, the measure of hypertrophy proposed by Cruveilhier for the right ventricle,—any thickness exceeding four or five lines,—is even wider from the mark than that proposed by him for the left; for, "in the male subject, the maximum thickness of the right ventricle, from the fiftieth to the seventy-ninth year, was only $2\frac{1}{9}$ lines, and in the female $1\frac{1}{4}$ lines," (p. 290.)

The walls of both ventricles are thinner in phthisical subjects than in others. It becomes a question whether this gradual and proportional diminution of all the heart's dimensions in tuberculous subjects should be considered a morbid condition of the organ. We coincide with our author in thinking that it cannot be esteemed a disease, any more than the atrophy of the muscles of animal life in the same individuals. Indeed, the coexistence of cardiac disease and phthisis seems very rare. Of fifty-seven tuberculous patients, not one suffered under functional disease of the heart. Even the œdema of the inferior extremities, observed in a certain number of them, was in every instance explained by the presence of organized coagula in the femoral and external iliac veins.

The same remarkable tendency to increase of size exists in the orifices of the heart as in its substance: they become wider as the subject advances in age. This fact is proved by our author's twelfth and thirteenth Tables, which also confirm M. Bouillaud's observations as to the excess of width of the right openings over the left. M. Bizot's researches, however, show that the difference between the sides is much greater than M. Bouillaud supposed. Thus, the mean circumference of the right auriculo-ventricular orifice exceeds that of the left by nine lines in the male, and seven in the female. The excess of the pulmonary orifice over the aortic is still greater, and is most marked, in the female subject.

The elaborate tabular exposition of the caliber, both absolute and proportional, of the various arteries, is too extensive for our pages. We must content ourselves with the short extract which follows, from the author's summary.

> "Hence it appears that, contrary to the general opinion, infancy is not the period of life at which the arterial system is of relatively the largest capacity. It attains its highest degree of development, both in diameter and thickness, towards the close of life.* The body, having grown for a time in every part, reaches the point at which all its constituent organs are accurately balanced in respect of their development. A retrograde action then begins. The muscles fall into a state of atrophy, the skin

* This is a new proof of the universality of the law discovered by Louis, that membranous tissues thicken in proportion to their dilatation. It was this law, established for the œsophagus, stomach, intestines, ureters, &c., that induced that philosophical observer to doubt the occurrence of what Corvisart termed passive aneurism of the heart. The progress of our knowledge in cardiac disease has, as is well known, proved the justness of his doubt, by showing the extreme rarity of such a morbid state.

wrinkles, the sensations lose their acuteness,* the intellect fails; all gradually die. A single system of organs continues to grow—*the heart and arterial system.*"

In making known this law of the vascular system, which overturns such an abundance of theories on growth, reparation, &c., M. Bizot has rendered a most important service to the science of general anatomy.

In order to ascertain the form of the arterial tube with exactitude, our author measured the width of *two thousand one hundred and sixty-two* vessels, at different points of their course. The various shapes observed, and their proportional frequency, were the following:

| | | |
|---|---|---|
| 1. | A cone, with its base turned towards the heart . | 858 times. |
| 2. | Two truncated cones, opposed at their truncated summits, | 610 |
| 3. | A cone, with its summit turned towards the heart . | 319 |
| 4. | A perfect cylinder . . . . . . . . | 248 |
| 5. | Two cones, opposed at their bases . . . . | 127 |

A fact which must forcibly strike the reader on a glance at the above table is, that the cylindrical, instead of being, as writers inform us, one of the most common, is, on the contrary, one of the rarest forms. Each vessel affects a certain form, which it presents much more frequently than any other. The figure of the popliteal, for example, is fusiform, or that of two cones opposed at their bases; a modification rarely found in any other vessel. It is worth considering whether the frequency of popliteal aneurism may have any relation to this fact.

The second part of the essay is devoted to the consideration of Structural Changes. Previously to entering on this branch of his enquiries, M. Bizot says a few words on a question of importance,—the pathological value of red coloration of the internal surface of the arteries. His experience has convinced him that such coloration, when unattended with thickening of the internal membrane, change of consistence, or any other morbid appearance, cannot be considered a morbid state. The results obtained by Trousseau, Louis, and Andral, are thus confirmed, and the fallacy of the notions entertained by Frank, Bouillaud, and others, still further proved.

M. Bizot has met with but one species of structural change of primitively acute form. It consisted of an exsudation on the internal surface of the vessel, varying in thickness, albuminous in appearance, and of the same consistence as a viscid jelly; perfectly transparent, smooth, either colourless or of rosy hue, and always unattended with injection of the subjacent or surrounding membrane. He conceives that all doubt as to this matter being the primary form of the white patches described in works by the term cartilaginous, is removed by the following considerations: Close to the above exsudation, or in some other point of the vessel, are found productions differing from it simply by their closer adhesion to the lining membrane; further on, the albuminous substance is more consistent, has a milky tinge, and adheres still more firmly to the arterial surface; lastly are observed whitish patches of different sizes, resembling in consistence and colour hard-boiled white of egg, or as dense as

* Perhaps the exception claimed by Bichât for the sense of *taste* may be admitted. He certainly did not much exaggerate in terming that sense "le dernier fil auquel tient le bonheur d'exister."—*Vie et Mort.*

cartilage. All traces of the internal membrane are now lost, and the adventitious patch is in contact with the middle coat, or connected to it by atheromatous matter. It follows from this that those authors are in error who describe the cartilaginous patches to be contained between the middle and internal coats of the vessels, instead of on the surface of the latter. M. Bizot also rejects the prevalent notion that the osseous scales formed in the arteries are transformed cartilaginous patches: his reason for doing so is that, according to the hypothesis of transformation, the transition from the cartilaginous to the bony state should be traceable; whereas, the most laborious research failed in showing the least appearance of ossification in the patches alluded to.

M. Bizot informs us that the albuminous matter, when secreted in isolated patches, does not cause any local or general derangement of function. The case is, however, widely different when an extensive surface is affected. Under these circumstances, a train of formidable general symptoms is the result. In confirmation of this fact, he gives the history and dissection of three cases of the affection. In these we discover a uniform course of symptoms; acute œdema, commencing by the inferior extremities, and thence spreading over the entire body, accompanied with fever, without any symptoms referrible to the heart or principal vital organs. The lesion found after death was the same in the three cases: an albuminous false membrane lining the whole internal surface of the aorta. We agree with the author in thinking that these cases are examples of acute aortitis.

The lesions of primitively chronic form are subdivided by M. Bizot into those common to the entire arterial surface, and those confined exclusively to the vessels of the extremities.

Every one who has attentively examined the surface of the aorta has often observed numerous specks, of the size of a grain of sand, of whitish yellow colour, unattended with the slightest redness in the adjoining tissues, and especially occurring in the neighbourhood of the coronary, carotid, subclavian, and intercostal arteries. Isolated in some places, they are in others united into groups forming yellow patches, and are evidently contained between the middle and internal coats; for, on removing the latter, part of the patch is found attached to it, while the rest adheres to the former. These patches go through a series of transformations, classed by M. Bizot under the two heads of *ulcerative softening* and *ossification.* He proves, by the closest reasoning, that the various lesions described under the names of pustules, abscesses, atheromatous, steatomatous products, &c., all spring from these almost invisible specks, and are developed without any trace of inflammation. He clearly shows that the term *abscess* is improperly applied in these cases of ulcerative softening: though the softened matter has some resemblance to pus, yet its mode of formation differs totally from this, and assimilates it much more to tubercle.

Morgagni and Haller long ago described the series of changes above alluded to, but they erroneously included the cartilaginous patch among the transformations of the yellow patches. Their account of the bony depositions also differs from M. Bizot's. According to him, the osseous formation commences by a small, hard, semi-transparent point, attacking at first the superficial layers of the middle coat, and gradually reaching the deepest, though rarely destroying that membrane altogether; while the internal membrane, after a time, completely disappears: and he maintains that this bony development "is, properly speaking, not a *transformation* of the spots, as Haller and Morgagni supposed, but that the yellow spots are in reality the *nidus*, in which the phosphate of lime is deposed," (p. 334.) He does not attempt to trace out the circumstances which severally cause the primitive lesion to end in ulcerative softening or the formation of bony matter.

The atheromatous matter, which we have already noted as being frequently formed under the albuminous patches, when in the cartilaginous state, sometimes undergoes the same transformations. Occasionally its abundance produces remarkable effects. In the smaller vessels, it causes obliteration of their cavity; in the larger, it destroys the middle membrane. The walls of the vessel so attacked lose their elasticity, from a double cause,—the destruction of the fibro-elastic membrane, and the growth of cartilage devoid of elasticity on their surface. Wherever this occurs, the vessel becomes an inert tube; a fact by which the dilatations of the aorta, without aneurismal tumour properly so called, have been plausibly explained.

Of the structural changes peculiar to the vessels of the extremities, our author describes two varieties. By comparing the following summary of his remarks with the account we have given of the other form of ossification, the delicate distinctions between them may be traced: In one of them the middle coat is primarily affected, and undergoes one transformation only,—the gradual solidification of its fibres; which at the same time grow transparent, and thinner than the original membrane. In the other, which is of less frequent occurrence, the middle coat itself appears to escape attack, while the ossified points are of granular shape, arranged in the form of an ellipse, and are not, in the outset, covered by the internal membrane.

The third part of the essay opens with some considerations on the Pericardium. The white spots so frequently observed on this membrane are of two kinds: of these, one species is produced by the transformation of the matter thrown out in inflammation on its external surface; the other, unknown in its exciting cause, is brought about by slow alteration of the membranous tissue itself, which gradually loses its normal transparence and thickness. The spots of the latter species are much more common than the former; three times more frequently observed in males than females; grow at a later period of life in the latter sex; increase in size and frequency of occurrence in the ratio of the age of the subject; are most commonly met with on the anterior surface of the right ventricle; and are uninfluenced in their origin or progress by the tuberculous affection. Such, at least, are the laws respecting them which M. Bizot's facts allowed him to deduce.

Relatively to fatty deposition under the pericardium, our author ascertained that its well-known greater frequency in females cannot be ascribed to their being usually fatter than the other sex: "for, of twenty-nine women without a trace of subcutaneous fat, fourteen had the heart surrounded with the maximum quantity of adipose tissue ever observed; while, of thirty men, equally spare in the body, three only were at all remarkable for fatty pericardium." Further on, we are told that, in six out of eleven phthisical men, there was a complete absence of fat under the membrane spoken of, and a very small quantity in the remaining five. The maximum of fat was found in eleven out of twenty-five tuberculous women; a moderate share in eleven others; while it was completely wanting in three. M. Bizot also found the heart itself fatty in eight subjects out of fifty-seven: all these eight were females, and died tuberculous. Connecting these remarkable facts with what is already known on the subject of fatty liver, the reader will not fail to perceive that they furnish valuable materials for investigating those strange aberrations from the normal state of the nutritive secretions observed in phthisis.

It is well known that Bichât, in his General Anatomy, guided by the difference of appearance observed, after a certain period of life, in the internal membrane of the venous and arterial systems, gives it as his opinion that they are dissimilar in original structure. M. Bizot rejects this doctrine because, 1st, in infancy, the two membranes are in every respect perfectly alike; 2dly, the ossifications of the arteries do not, as Bichât supposed, originate in its lining membrane; 3dly, the venous membrane, though very rarely, is occasionally affected in the same manner as the arterial. In forty-four pulmonary arteries, M. Bizot detected spots nine times. The reader to whom the researches of the modern French school are familiar will feel no surprise in learning that, in forty-four out of 155 subjects, M. Bizot found the foramen ovale permanently open, and in every case unaccompanied with symptoms of cardiac disease. Former observers had distinctly proved the fallacy of the old doctrines concerning the connexion of cyanosis with this malformation.

It appears, from a table showing the periods at which the spots and ossifications were first detected in each artery, from the earliest age upwards, that a remarkable difference exists in the time of life at which these lesions are habitually developed in any two given vessels. Thus, as regards the simple spots, the primitive iliacs were the earliest affected in our author's cases, and this at the age of seven; while no similar lesion was found in the brachial artery before the age of forty-eight; thus making a difference of thirty-one years between these vessels. They occupy the two extreme places in the scale. An analogous distinction exists with respect to ossification: the first bony plate met with was found in the posterior tibial arteries of a subject thirty-five years old; while in the internal carotid no trace of similar formation was detected earlier than the sixty-sixth year. The other vessels take intermediate places in the scale. Another important fact disclosed by this table is, that the vessels nearest the aorta are those first attacked by the *yellow spots*, while those farthest from it are the earliest to present *osseous deposits*. Among the physiological inferences drawn by M. Bizot from these data is an ingenious mode of accounting for the force and hardness of the pulse in old subjects.

"A great part of the arterial system," he says, "having lost its elasticity in individuals of advanced age, from the development of organic changes, the entire force of the heart's action—which would be partly spent in dilating the whole system of arterial tubes, had they been all elastic,—is transmitted to whatever vessels remain sound. Now, as the radial is among the latter, it is perfectly natural that the pulse should be stronger in the old than in the young," (p. 393.) . . . . "Another probable consequence of this want of elasticity is, that bloodletting must weaken much more in advanced age than in youth: for, as the arterial coats do not return on themselves in old subjects, it follows that, by removing a certain quantity of blood, we take away from that which remains a considerable part of the sum of pressure exercised on it by the large vessels. Now, this effect can only be produced to much less extent in youth, inasmuch as, from their great elasticity, the walls of the vessels adapt themselves to the diminished quantity of fluid, and continue to compress it." (p. 394.)

By comparing the different parts of each vessel with respect to the frequency of their lesions, M. Bizot ascertained that they occur most commonly in the neighbourhood of the origin of other arteries, and especially of large trunks.

The essay terminates with an account of a most interesting law of morbid development, called by its discoverer *the law of symmetry of lesions.* On inspection of the tables introduced into the work, we find a close similarity in the figures indicating the number of morbid changes found in any given artery on the right and left sides. But, further, when each pair of vessels was studied carefully in a series of *individuals*, the exact resemblance of the right and left lesions in each subject, not only as respects their number, but their exact position, proved the law in question one of the most general of morbid anatomy. It is true that to it, as to every other law, there are exceptions; but they are few in number, and, for the most part, easily accounted for. M. Bizot assigns three reasons for the occurrence of exceptional cases. These are—the change of structure not commencing simultaneously at both sides; the want of symmetry in some of the vessels themselves, as, for example, the common carotid and subclavian arteries; the nature of the lesions, the albuminous patches being less under the influence of the law than the other morbid products. Now, as the yellow spots have a strong tendency to grow underneath the cartilaginous plates, the existence of the latter is a cause of absence of symmetry in the former. We cannot follow M. Bizot in his details relative to each vessel, but extract a few points from his summary. In 2171 cases of simple spots, symmetrical arrangement was wanting in only sixty-one instances. In 659 instances of consecutive lesions, (including those of inflammatory nature,) exceptions occurred fifty-one times only; and, if we exclude from these fifty-one, twenty-nine cases of albuminous or cartilaginous patches, there remain but twenty-two that do not acknowledge the law.

The reader cannot have failed to expect that the collation of our author's results with the various facts hitherto ascertained on the history of aneurism should illustrate the cause and mode of growth of those tumours. He himself has not neglected this view of his subject. The first point, well deserving the consideration of those who are disposed to include the various structural changes of the vessels among the causes of aneurismal disease, is the extreme frequency of the former compared with that of the latter. The result with respect to age, too, is remarkable. The ages of 108 subjects affected with aneurism, and ascertained from various authors, were as follows:

| | | | |
|---|---|---|---|
| From 10 to 19 . . . | 1 subject. | From 50 to 59 . . . | 14 subjects. |
| .. 20 .. 29 . . . | 15 .. | .. 60 .. 69 . . . | 8 .. |
| .. 30 .. 39 . . . | 35 .. | .. 70 .. 79 . . . | 2 .. |
| .. 40 .. 49 . . . | 31 .. | .. 80 .. 89 . . . | 2 .. |

Now, judging from this table, and from the fact that it was not at the time of the origin of the disease, but at that of the patients' seeking advice, these ages were noted, we must admit that the laws regulating the period of development of the two morbid conditions are almost the converse of each other. Next, passing to the influence of sex, we find that, of 189 subjects affected with aneurism, 171 were men, and only eighteen women; while M. Bizot's tables of arterial lesions do not in any case give such a majority to one sex over the other. If the law of symmetry be taken into consideration, a new point of discrepancy is discovered; for, of 138 subjects labouring under aneurism of the extremities, six only were affected with symmetrical tumours. Lastly, it is well known that non-traumatic aneurism of the limbs is much more frequent in the popliteal than in any other artery. M. Bizot found seventy-five cases of popliteal among 145 aneurisms. Yet, on the other hand, that vessel is far from being the most frequently affected with consecutive lesions, occupying in this respect only the seventh place in the scale. However, the quantity of movement to which the popliteal artery is exposed must be taken into account, as well as the fact that the posterior tibial artery holds the *first* place in frequency of structural lesions, and that many aneurisms, called popliteal because seated in the ham, may (as our author suspects) really belong to the origin of the former vessel. Be this as it may, however, the general force of these considerations seems to warrant the conclusion drawn from them by the author: "Although there be strong reason for admitting that lesions of the vessels, and even 'occasional causes,' are of great importance in the etiology of aneurism, yet there must exist some other cause beyond these, the nature and mode of which are unknown, and still need deliberate investigation." (p. 411.)

We cannot close our sketch of this valuable memoir without confessing that we have by no means been able to do it justice. It may truly be said of it, as of Haller's Physiology, that each line contains a fact. Enough of its sterling matter has, we trust, nevertheless, been transferred to our pages to induce the reader to study the original. If its perusal have no other effect, it will at least convince him of the vastness and dignity of medical science. Of the host of writers who have taken the arterial system for their theme, how few have done more than glance at any of the points investigated by M. Bizot; and yet how much has even he left undone? And what can be better calculated to elevate our notions of a science than to prove that the pettiest points connected with it may be invested with importance, when they are, in a multitude of cases, accurately established, examined, and compared?

III. *Essai sur quelques Points de l'Histoire de la Cataracte.* Par Th. Maunoir. (*Essay on certain Points in the History of Cataract.* By M. Maunoir.)

M. Maunoir, author of this memoir, is nephew to the celebrated Genevese oculist of that name. Familiarized by early education with researches on the eye,—trained for original observation under the guidance of Louis,—and animated with a strong desire of coupling his name with some useful and sound discoveries, M. Maunoir brought to his task the most important requisites for the investigation of truth; and the result of his labours is of that valuable kind which might have been anticipated where such elements of success existed.

The extensive hospital practice of Professor Roux supplied our author with a wide field for his enquiries. The celebrity enjoyed by that surgeon for his operations on cataractous eyes (whether their ordinary event justify it or not) brought numbers from all parts of France to La Charité: and, as he is in the habit of confining his operations for cataract to the spring and autumn, no better opportunity for studying the affection could be had than that afforded by his wards during those seasons. The number of cases on which M. Maunoir's researches are founded amounts to 121, collected in four seasons, giving a mean amount of thirty operations a season.

There is, perhaps, scarcely any human ailment better understood than cataract, as far as regards the nature of the disease, its principal symptoms and varieties, and the surgical means fitted for its cure. M. Maunoir was fully aware of this, but did not, in consequence, despair of adding to our *accurate* knowledge of the affection by bedside observation and the numerical method. He saw that the exact proportion in which a given symptom ordinarily occurs, for example, and the side on which truth really lies where the opinions of first-rate men are at variance,—(let us instance the comparative excellence of extraction and depression,)—might be discovered by their help, and by it alone. In fact, with respect to the history of this disease, as of every other, though in a less degree than is the case with many, there are many lacunæ to be filled up, no few errors to be rectified, and, in almost every instance, positive values to be substituted for vague approximations.

Our author commences his analysis with an enquiry into the influence of those agencies generally looked on as *causes* of the disease. Sex, in the first place, cannot be said to affect its development; for, of his 121 patients, placed in wards containing an equal number of beds for each sex, sixty-one were males and sixty females. This confirms a statement, published some time since as the result of an examination of some cases of Dupuytren, 207 in number: of these, 135 were males, seventy-two females—a proportion exactly coinciding with M. Maunoir's, as the number of beds in Dupuytren's men's ward precisely doubled that in the women's.

Our author's tables give a good idea of the influence of age in causing opacity of the lens. The fourth or fifth of the total number of cataractous subjects is found between the ages of fifty and sixty; the third between sixty and seventy; a little less than the fourth beyond that latter age. These proportions, which, of course, refer to the period when the patients applied for assistance at the hospital, are confirmed by his results respecting the time of commencement of the disease.

In regard to this statement, it is, however, important to observe that, in making calculations as to the comparative frequency of occurrence of cataract at different ages, it is of essential importance to distinguish the particular forms. In early life, opacities of the lens or capsule, or both together, occur; but, for the most part, under pathological conditions differing from those which are found to hold in after-life. Capsular cataract also may be connected with a very different condition of the eye from lenticular. In aiming at accuracy, it is therefore not enough to say that, of a certain number of cases, so many occurred between fifty and sixty, and so forth; but we ought, first of all, to describe the exact form of these cataracts,—whether lenticular, or capsular, or capsulo-lenticular, and whether with or without complication.

It is impossible to doubt, from the evidence of M. Maunoir's cases, that cataract is, to a certain extent, an hereditary affection. Hereditary influence was, indeed, traced in upwards of one-fourth of the individuals examined: and, with respect to cataract, there are none of those especial difficulties in establishing such influence which in other cases interfere with the validity of our apparently just conclusions. It is not, for instance, one of those affections, such as phthisis, which are so extremely common that, without any tendency being inherited from parents but from the simple fact of their habitually attacking a large body of the population, they must frequently occur in several members of the same family.

"Of thirty-nine subjects (twenty-two male, seventeen female,) who were enabled to give certain information on the point in question, twenty-two affirmed that their parents had never had the slightest complaint capable of giving rise to a suspicion of cataract. Ten others, on the contrary, (four men, six women,) assured me that one or more members of the family had suffered from the disease. In almost every instance the operation had been performed, so that there could be no question of the correctness of the diagnosis." (p. 79.)

Various other points, usually examined by writers in their etiological chapters, are passed in review by M. Maunoir: nor has he failed to come to positive results respecting them. Thus, he found individuals following certain trades more frequently affected with cataract than others; and certain habits of living, constitution, &c., coexisting with the disease in larger proportion than others: but he wisely abstains from concluding thence that the various circumstances alluded to should be numbered among the *causes* of cataract.

The commencement of the disease (a point of its history requiring examination, perhaps, more than any other,) receives tolerably full investigation from M. Maunoir. In the majority of cases, cataract commences slowly; the patient distinguishes objects, as it were, through a mist, and the morbid condition is confined to one eye. Such was the course of the affection in *fifty-two* cases out of *sixty-two:* in the other ten subjects, (eight men, two women,) the sight was lost more or less suddenly. In one instance, the patient went to bed without his visual powers being in the least impaired, and in the morning was scarcely able to guide himself; both eyes being equally affected. In *sixty-three* cases out of *seventy-two*, the disease commenced in one eye; the right being the first attacked *thirty-five* times, the left *twenty-eight*. In *nine* subjects, the faculty of vision became weakened in both eyes simultaneously. In *eight* out of *seventy-two* patients, the use of one eye was even completely lost before they perceived its being affected at all. The discovery of its condition occurred from accidentally closing the sound organ.

"May cataract often remain confined to *one* side during a series of years, or even for a whole lifetime; or, once developed in either eye, are the chances extremely strong that the other shall be similarly affected within a certain time? On first thought, this question may appear one of mere curiosity, but a little consideration will show its practical interest. If it were proved that the formation of a *second* cataract may be prevented, or that, when in its commencing stage, a spontaneous cure may be induced by simply operating on the eye first affected, the importance of rejecting the rule of practice usual in such cases—not to operate until both eyes are affected—becomes manifest." (p. 89.)

M. Maunoir is aware that the spontaneous cure of cataract is doubted by the majority of surgeons, but contends that they are rarely placed in circumstances suited for ascertaining the fact with precision. Besides, all writers do not agree on the point. Wenzel and Saint-Yves have both related cases of complete restoration of sight in persons who had only one lens operated on, though affected with perfect double cataract.

The opinion current in the profession respecting the latter question is, that, in the majority of cases, when the disease seizes one eye, the other ends by being similarly affected. This opinion has grown out of the great predominance of cases of double cataract observed in the hospitals, and even among the better classes. The conclusion is, however, not fairly drawn from the premises. Is it not quite as likely that the greater number of subjects presenting themselves for treatment with double cataract may arise from the fact that persons who continue to see with one eye refrain from seeking surgical aid? In this way, cases are met with of individuals that have been affected with single cataract for years without thinking of its removal. Why, in the same manner, it may be said, should not opacity of one lens exist in a number of persons during their *whole lives*, without their taking steps for its treatment? Spite of this rather plausible reasoning, M. Maunoir is induced to conclude the vulgar opinion to be in the main correct. His reasons for doing so are logical and forcible.

"First. In nine subjects out of seventy-two, the disease commenced in both eyes at the same time. 2dly. Eight only of these seventy-two individuals had completely lost the use of one eye when they detected a weakness of sight in the other. Hence there were only eight cases in which we are justified, strictly speaking, in supposing the first cataract to have existed *alone* for any considerable time: but even this is neither proved nor, from what follows, likely. 3dly. In every other case, the second cataract commenced growing a few weeks, months, or at the most from three to five years after the first. In two persons only was this period extended to ten and twenty-five years. 4thly. The mean space of time, in forty-seven cases, between the commencement of the disease and the period at which the patients grew so blind as to require a guide, was five years and a half." (p. 92.)

In whatever way this question be ultimately settled, the extreme frequency of double cataract is a remarkable phenomenon. Nothing, as our author remarks, is more rare than to see cancer attacking *both* breasts or testes: yet there is as complete a similarity in structure and functions in the case of those organs as of the lenses; and, as far as we can judge, the cause of cancer, when once brought into action, is as deeply rooted in the economy as the cause that produces cataract. This peculiarity is not, however, by any means confined to cancerous disease. Acute affections present it also; and, indeed, it seems to be more closely connected with the nature of the tissue than of the morbid action. Thus, Louis has shown that *double* pleurisy is almost unheard of, except in cases of organic affection of the lungs; pneumonia rarely becomes double,—we have found it so in only forty instances out of 321 cases, collected from various authors,—and is almost never so primitively; there are but two or three authentic cases of double non-calculous nephritis on record; while, on the other hand, in *sixty-two* out of *seventy* patients affected with tonsillitis, and observed by Louis and Rufz, both amygdalæ were inflamed together.

M. Maunoir's chapter on the Symptoms gives evidence of patient enquiry; and we agree with him in attaching importance to the study of the anomalous visual sensations occurring in cases of blindness, from whatever cause it may arise. A close acquaintance with them might very possibly often clear up a doubtful diagnosis, where the examination of the organs themselves, as too often happens, had failed to give absolute certainty. There is little, however, sufficiently novel in its contents to repay the trouble of extraction. In detailing the state of the pupils, and the degree to which the power of seeing was retained by his patients, M. Maunoir relates cases distinctly proving that immobility of the iris, irregularity of the pupil, and almost total incapacity of distinguishing light from darkness, are insufficient to warrant the surgeon in diagnosticating amaurotic complication.

Relatively to their appearance before extraction, cataracts are divided into four classes. Of 115 lenses removed from sixty-four patients, the following was their relative proportion:

| | | | |
|---|---|---|---|
| Gray . . | 47 | Blueish . | 22 |
| White . . | 41 | Blackish . | 5 |

In addition to its colour, the uniformity or striated appearance of the cataract is worth attention. The latter character is not rare: it existed in twenty-six of the sixty-four subjects alluded to, most frequently on one side, seven times only on both. The early writers on the disease attached great importance to the colour and uniformity of aspect of the lens. Maître-Jean, taking them as guides in prognosis, drew up a list of favorable and unfavorable colours. In more modern times this view of the question has been abandoned; but the generality of oculists affirm, as is well known, that, from the appearance of the cataract, we are enabled to determine if the opacity be capsular or lenticular, soft or hard. Thus, Beer and Travers assert that *membranous* cataract is always bright in colour, and of spotted or variegated appearance; never uniform. According to the former ophthalmologist, again, the tint of *caseiform* cataract is never uniform, but always maculated, the spots resembling fragments of mother-of-pearl. The opinion of Travers on the latter point is to precisely the contrary effect. In order to test these opinions by accurate observation, our author commences by enquiring into the frequency of capsular cataract. If we take the general sense of writers on the question, we must consider it almost as common as the lenticular variety. Dupuytren goes even further. He declares that the membranous occurs in the ratio of 1 : 1½ to the ordinary species: yet, on referring to a table of 264 operations for cataract, (eight only by extraction,) performed by that Professor, we find but fifteen noted as membranous, which gives a proportion of 1 : 16⅗! M. Maunoir, in order to account for this monstrous difference, suggests the probability of an error of impression,—an explanation of the difficulty which is hardly defensible, as the same statement is repeated in two works published under Dupuytren's own eye. Turn we to our author's cases. In these, *extraction* was performed 179 times, and in *five* cases only was the capsule evidently opaque. Here we have a proportion of $1 : 34\frac{4}{5}$ between the varieties in question. In six other cases the pupil was not perfectly clear after the lens had been removed; but in these instances the trifling opacity observed depended quite as probably on some fragments of the lens remaining *in situ* as upon commencing opacity of the capsule. Granting them to have been capsular cataracts, however, the proportion is still as $1 : 15\frac{3}{11}$. Now let the reader bear in mind that M. Roux's operations are all by *extraction*, and that, consequently, error on the question at issue was impossible on the part of M. Maunoir; whereas Dupuytren's cases were, with eight exceptions, couched, and consequently himself liable to be deceived respecting the number of membranous cataracts among them. We need not adduce any stronger evidence of the error of Travers's and Beer's diagnostic instructions than the facts just learned from our author's experience. We have only to refer to them to see that, of 117 cataracts, five only were evidently capsular, while thirty-three had the maculated surface in question. In one only of these five cases had the opacity of the capsule any peculiar or distinctive appearance: in this instance a small white square spot, situated on a brownish base, presented itself in the centre of each pupil.

Beset with difficulties as enquiries respecting the duration of chronic diseases usually are, those relating to cataract are especially so. Many individuals are so utterly careless about themselves that they take no note of what perhaps can alone be looked to with any confidence as a guide in the investigation,—the period when weakness of sight commences. Nor, it must be confessed, can we be certain that the time of origin is thus faithfully indicated, where patients have been watchful enough to notice it. Similar sources of error belong to other diseases. Among those peculiar to cataract is the fact already alluded to, that one eye may be affected for a considerable time before the individual discovers its condition, which he eventually does by chance. Nevertheless, these and other obstacles, which it is needless to enumerate, do not render the final settlement of the question impossible, but simply increase the number of cases; and hence the time necessary for its elucidation. M. Maunoir's results on the subject are derived from the histories of forty-seven patients, the only individuals, of those examined by him, on the accuracy of whose statements he could fully rely. They are as follows:

"1st. (*No distinction of sex being made.*) The mean duration of the disease from the moment of origin, as far as this was determinable, to the time at which the patients ceased to be able to go about without a guide was about five years, one month, and four days. 2dly. In thirty out of thirty-two cases the cataracts on both sides occupied a different time in reaching maturity; and in twenty-one of these thirty cases the progress of the disease in the eye first attacked was the slower. 3dly. The mean period of maturation for the eye first affected was two years, five months and a third; for that last attacked one year, eight months and a half. 4thly. (*The sexes being distinguished.*) The total duration of the disease, as well as of *each* cataract, was greater in females than in males. 5thly. The second eye remained perfectly healthy for a shorter or longer time after the first was lost, in thirteen men out of eighteen, and in seven women out of fourteen; and, generally speaking, that time was much longer in the former than in the latter. The disadvantage here observed on the side of females will be found to exist with respect to the prognosis also." (p. 118.)

Our author now passes to that portion of his subject which will doubtless appear of most importance to the majority of our readers: the operation, its difficulties, the accidents which may interfere with its completion, and its consequences. This chapter derives much interest from its containing the first analytical enquiry into the practice of the most dextrous operator on the eye of whom the Parisian school can boast, Professor Roux. Exclusively devoted as this gentleman is to the method by extraction, his cases necessarily furnish no data for solving the problem respecting the superiority or inferiority of that method, as a general practice, to couching. Considerable light is, however, thrown in the pages before us on several questions, which have long divided our best oculists; but, in the first place, it may be well briefly to sketch M. Roux's mode of proceeding in his cataract cases.

The period for operating, as we have said, is confined to spring and autumn. The patients, on their admittance into the hospital, are put on whey diet; mustard pediluvia are used daily, venesection being very rarely had recourse to. The morning of the operation a blister is applied to the nape of the neck, and kept open till the patient is permitted to leave the hospital. No means are ever, or scarcely ever, employed to dilate the pupil. If both eyes are in a fit state they are operated on at the same time. With respect to the operation, for the minute details of which we must refer to our author's description, the following particulars seem to require notice. It is always performed with Richter's triangular knife. The puncture of the cornea is practised considerably above the external extremity of the transverse diameter of the eye. The exact point varies: when the eye is favorably disposed, it is so managed that the incision is as much external as inferior. The point of the knife is brought out at such a point on the other side of the cornea, that the section, when completed, embraces a little more than the semi-circumference of that membrane. The section is made altogether by pushing the knife onwards parallel to the iris, and never by bearing from above downwards. So important does M. Roux consider this mode of dividing the cornea, that he constantly employs it, even at the risk of pricking the skin or conjunctiva of the internal canthus. This done, the operator introduces a *kystotome*, with blunt convex back and concave edge, and with it incises the double membrane investing the anterior surface of the lens. The usual means are then had recourse to for the gentle expression of the latter body.

The dressing applied after the operation, consisting of a white linen compress, followed by a black silk bandage, is removed for the first time on the fifth day. From that time the patients are directed to bathe the eye frequently with cold or lukewarm water, whitened with a few drops of solution of acetate of lead. On the first day they are left completely without food; from the second to the fifth, they are given broth; then soup; and lastly, the half or three quarters of the hospital allowance.

There is no ward set apart for ophthalmic affections at La Charité. The beds of the patients operated on are, as a substitute for that convenience, surrounded with thick sheets to exclude the light. In consequence of this plan, there is an exceedingly imperfect circulation of air about the patient; and M. Maunoir, with much plausibility, conjectures that some cases of failure of the operation may, in a great measure, be ascribed to the fatigue and restlessness resulting, in warm weather especially, from this circumstance. There is another incommodious arrangement at La Charité, adverted to by our author, but which affects the female patients only. From the position of the women's ward, they are obliged, after leaving the operating theatre, to ascend a staircase exposed to strong currents of air, in which we have ourselves frequently shivered, before reaching their beds. It is the more extraordinary that M. Roux should allow these drawbacks to the success of his operations to continue to exist, as the chief reason we have heard given for his confining his time for extracting to the spring and autumn is the greater evenness of temperature at those seasons.

The only difficulty alluded to by M. Maunoir, as occasionally thwarting the operator, arose from the extreme mobility of the eye in some cases. Many patients are, as is well known, totally unable to control its movements. He suggests a feasible method of preventing the occurrence of this obstacle; namely, directing the patient to practise, for some days previous to the operation, holding the organ steady. We are not aware whether such a plan has yet been made trial of.

The errors and accidents observed during the operation were the following:

"1st. The section of the cornea was too small in one case to allow of the passage of the lens; it was necessary to enlarge it with scissors. 2dly. In some cases the operation was not finished from either of the following causes: *a*, after the introduction of the knife into the eye, a sudden movement of the patient caused it to slip out; *b*, a similar movement drove the point of the instrument through the iris or pupil into the posterior chamber, withdrawal of the knife being followed by evacuation of the aqueous humour; *c*, the pressure of the upper eyelid, intended to express the lens, caused the escape of some of the vitreous humour, while the cataract remained *in situ*. 3dly. The vicinity of the internal canthus was rather frequently wounded, at the moment of completing the incision of the cornea. 4thly. The iris was wounded a certain number of times under various circumstances. 5thly. A portion of the vitreous humour escaped either before or, as more frequently happened, after the expression of the lens." (p. 125.)

Here is abundant encouragement, it may be said, for our young surgeons: if such be the feats of the first operator in France, few of them need tremble for their reputations. And yet it must be admitted that the escape of some vitreous humour is not uncommon with every operator, nor is it, within certain limits, detrimental. Wounding the iris, or even cutting away a piece of it, is not deadly. These and similar accidents arise in some degree from the nature of the material we have to operate on, and cannot be avoided even in the best hands—in the hands of better oculists than Roux. The young surgeon who should begin to operate without being perfectly aware of these accidents, and without having seen them occur, would be to blame, because he would not be prepared for them when they did occur.

Independently of these blunders in the operation, certain other accidents frequently supervene after it, and mar its success. They are either local or constituted by intercurrent diseases totally unconnected in their origin with the condition of the eye: and, indeed, it is remarkable that the complete absence of morbid phenomena after the operation is extremely rare, even in cases of perfect success as regards the restoration of sight. Thus, of twenty-five patients in whom the operation was done on both sides, and twenty-six on one side, five only were altogether free from morbid symptoms during the progress of the cure. M. Maunoir devotes an extremely instructive and interesting chapter to the various phenomena observed in such cases; one of the most remarkable of which is a pain affecting the teeth, and apparently seated in the branches of the fifth nerve.

In estimating the results of operations for cataract, M. Maunoir acquiesces in Professor Roux's opinion as to the necessity of making a distinction between the eye itself and the individuals affected with cataract. Now the chances are always more in favour of the latter than the former; that is, there are proportionally more patients operated on who recover their sight than eyes on which the operation proves successful. From our author's experience, the following is the ratio between them: Of 115 patients operated on by extraction, either on one or both sides, seventy-three recovered their sight through the operation; or a little more than five out of every eight operated on. Of 179 operations, ninety-seven were successful, or a little less than five in nine.

The different lesions to which loss of sight was attributable in cases of failure may be comprised in the following statement:

"1st. Fourteen eyes were destroyed by suppuration. 2dly. In nineteen cases, the cornea become so opaque that nothing could be distinguished behind it. 3dly. In nine cases, where the opacity of the cornea was not perfect, an opaque point was detected behind it in the field of the pupil. 4thly. There were fifteen cases of *membranous* cataract, unaccompanied with opacity of the cornea or deformity of the pupil. In three or four instances, these cataracts had an evident rosy tint, of perfect uniformity. 5thly. In six cases, there was an opaque body in the field of the pupil, with displacement or marked deformity of that orifice. 6thly. Complete occlusion of the pupil occurred in one case. 7thly. There remain eighteen cases that cannot be classed with the above: in one of these, though both pupils were perfectly black, the power of seeing was totally abolished."

In three or four cases only were symptoms of iritis noted: however, M. Maunoir could not affirm that they did not occur oftener, as he unfortunately neglected in some patients to observe the state of the iris. We are of opinion that, if M. M. had observed these cases more closely, as he ought to have done, he would have found many more instances of iritis. Jäger, of Vienna, is of opinion that some degree of iritis occurs in every case of extraction.

It appears from this summary, that membranous cataract existed in upwards of the third part of the unsuccessful cases. Such a proportion appears directly at variance with M. Maunoir's former conclusions respecting the rarity of that form of disease. This apparent contradiction, however, is easily explained. "For," says M. M., "I formerly spoke of cataract in general, and not of the patients who undergo unsuccessful extraction at La Charité. I have no doubt that their frequency in the latter subjects results from the mode of operating adopted by M. Roux. Is it not evident that, when a simple incision is merely made in the anterior part of the capsule, that membrane may become opaque, inasmuch as it remains in the eye; whereas, such a result would be impossible were it cut into pieces, as Beer advises, and brought out of the eye, in part at least, along with the lens?" (p. 146.)

We close our notice with a brief view of our author's valuable conclusions respecting the prognosis of the affection.

1. The age of the patients did not appear to affect the result of the operation from the twentieth to the sixty-ninth year; beyond that period of life, its effects were less favorable than at an earlier one. 2. The instances of success were somewhat less numerous in females than males, whether the eyes or the individuals be considered. 3. From a comparison of forty-eight simple with sixty-four double operations, it appears the results obtained by operating on one eye were less advantageous than those derived from operating on both organs the same day. 4. Slight puncture of the internal canthus, observed twenty-seven times, had no bad influence on the event of the operation: of these twenty-seven cases, sixteen terminated by recovery of sight. 5. The effect of lesion of the iris was not more detrimental; for, in twenty-one cases of this accident, eight only proved failures. The quantity of blood effused was not sufficient, in any instance, to prevent the operation from being completed; sometimes not a drop was spilt. 6. The escape of a portion of the vitreous humour, on the contrary, is one of the most formidable mischances that can occur; for, of nineteen cases in which it took place, six only terminated favorably.

It is not a little remarkable that the two last propositions should be in direct opposition with the opinions usually maintained by writers. M. Maunoir's are the first results that have been obtained by accurate and numerical analysis on the questions referred to: to them, therefore, we give our adhesion in preference to the vague recollections on which the contrary statements are founded. M. Maunoir does not mention the quantity of vitreous humour lost, which is a matter of some importance: moreover, it must be remembered that there are certain states of the vitreous humour in which it escapes more readily than when perfectly healthy; these states of the vitreous humour are always accompanied by a constitution of the eyeball not absolutely opposed, but not favorable to success.

Pressed as we have been for space in this review of M. Maunoir's essay, we had either of two courses to choose in drawing it up. We might have noticed a few of his results; examined at length their bearing on the doctrines of former writers; traced their probable influence on opinion and practice; or we might have given a fuller analysis of his work, allowing the sagacity of the reader to supply such considerations as we might in consequence have necessarily omitted. The latter is the mode of proceeding we fixed on, convinced, as we are, that it is the best calculated to give a fair notion of the labours we wished to make known to our readers; and of fitting them for usefully reforming such notions as required reformation. But, even in this way, we have been compelled to pass over in total silence a quantity of minor details which at once contain facts of high interest, and, from the conscientious diligence that characterizes them, furnish a wholesome lesson to the practical enquirer.

### IV. *Mémoire analytique sur l'Orchite blenorrhagique.* Par M. MARC D'ESPINE. (*Analytical Essay on blennorrhagic Orchitis.* By M. M. D'ESPINE.)

THOUGH not devoid of merit or interest, this production ranks infinitely below those with which it is associated: indeed, we question strongly the soundness of judgment evinced by the Society in allowing to appear, among its earliest labours, a work in which one of the very elements regarded by them as of paramount importance, namely, extensive and prolonged observation, is essentially wanting.

